# Single-molecule localisation microscopy approaches reveal envelope glycoprotein clusters in single-enveloped viruses: a potential functional role?

**DOI:** 10.1042/BST20240769

**Published:** 2025-06-18

**Authors:** David J. Williamson, Cecilia Zaza, Irene Carlon-Andres, Tobias Starling, Alessia Gentili, Joseph W. Thrush, Audrey Le Bas, Ravi Teja Ravi, Stuart Neil, Ray J. Owens, Maud Dumoux, Sabrina Simoncelli, Sergi Padilla-Parra

**Affiliations:** 1 Department of Infectious Diseases, King's College London, Faculty of Life Sciences & Medicine, London, U.K; 2 London Centre for Nanotechnology, University College London, London, U.K; 3 The Rosalind Franklin Institute, Harwell Science Campus, Didcot, U.K; 4 Division of Structural Biology, Nuffield Department of Medicine, University of Oxford, Oxford, U.K; 5 Randall Division of Cell and Molecular Biophysics, King's College London, London, U.K

**Keywords:** biophysics, envelope glycoprotein, fusion, microscopy, single molecule

## Abstract

Understanding how viruses enter and fuse with host cells is crucial for developing effective antiviral therapies. The process of viral entry and fusion involves a series of complex steps that allow the virus to breach the host cell membrane and deliver its genetic material inside, with viral fusogens often co-operating to attain the required energy for successful membrane fusion. This co-operative clustering of fusogens in viral envelopes is similar to receptor clustering in cellular systems, where receptors aggregate to initiate signalling cascades. Single-molecule localisation microscopy (SMLM) approaches have emerged as powerful tools to study these intricate mechanisms, allowing the observation of proteins with unprecedented levels of detail. These technologies provide unparalleled insights into the dynamics of viral entry and fusion at a molecular level, revealing how the co-ordinated action of fusogens facilitates membrane fusion. By employing the newest advances in SMLM techniques, such as DNA-PAINT and MINFLUX, we anticipate that precise information on the key steps of viral fusion can be revealed with high spatial and temporal resolutions, identifying critical points in the process that can be targeted by antiviral strategies.

## Introduction

The dynamic processes of cellular signalling and viral fusion depend on the orchestrated assembly of molecular machines, with receptor clusters and viral fusion proteins serving as key players. Both processes involve the precise aggregation of specialised proteins to facilitate critical biological functions [1-4]. In cellular signalling, receptors cluster together to amplify and propagate external signals, creating a robust and efficient communication network within the cell [5]. Similarly, viral fusion machines, or fusogens, must congregate at the host cell membrane to overcome energetic barriers and achieve membrane fusion [6], allowing the viral genome to enter and hijack the host cell machinery. These processes are fundamental to maintaining cellular homeostasis and enabling viral infection, respectively. Central to the viral-fusion process is the concerted action of fusogens, which must synergise to attain the necessary energy to merge two lipid bilayers into a single, continuous membrane. This intricate dance requires precise timing and spatial coordination to overcome the repulsive forces between the negatively charged membranes [7]. Lipids play a crucial regulatory role in both receptor clustering [8] and viral fusion [9]. The composition and fluidity of the lipid bilayer influence the distribution and mobility of receptors and fusogens, thus modulating the efficiency of these processes. Lipid microdomains, such as lipid rafts [10], can serve as platforms that facilitate the congregation of these proteins, thereby enhancing signal transduction and fusion efficacy. Understanding the interplay between lipids and these molecular machines provides deep insights into the fundamental mechanisms of cellular communication and viral entry.

**Figure 1:**
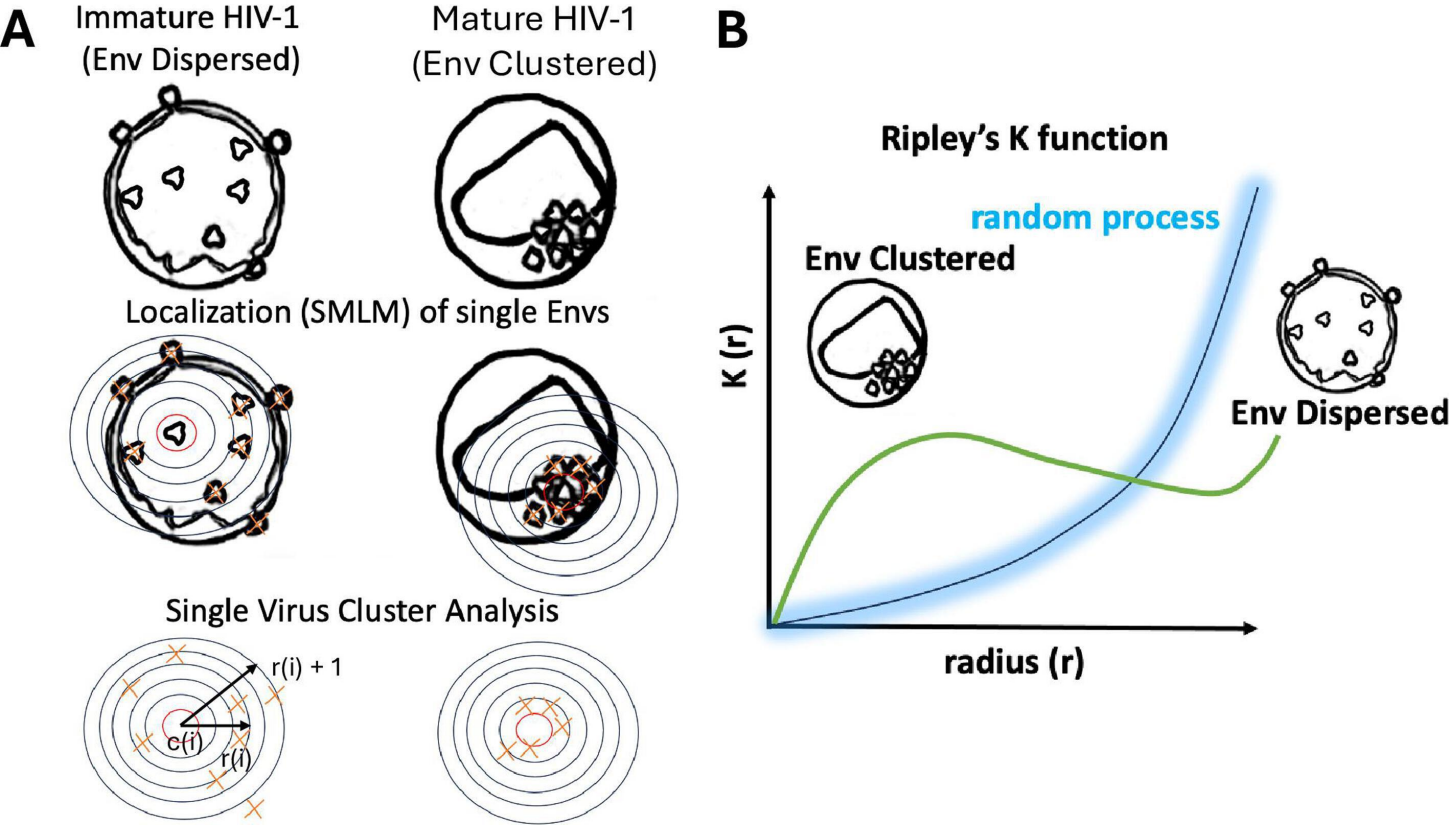
Clustering of Envelope Glycoproteins on Enveloped Viruses. (**A**) It has been shown by Chojnacki and co-workers [11] that in immature HIV-1 virions, Env proteins appear to be randomly or evenly dispersed across the viral membrane, whereas in mature virions, they tend to cluster, likely due to structural rearrangements in the viral membrane and interactions with the underlying Gag lattice. The localisations (red crosses) coming from immature (left column) and mature HIV-1 viruses (right column) can be plotted employing the Ripley function [12] to ascertain how far away these Env positions are from a random distribution. This approach permits to define what the meaning of Env microclusters: non-random distribution as seen for mature viruses. (**B**) Ripley’s K function is a spatial statistical tool that quantifies the distribution patterns of points, distinguishing between complete spatial randomness (random process, blue line), clustering (maximum green line), or dispersion (relative minimum, green line). In the context of HIV-1 envelope (Env) glycoproteins, this function can be applied to analyse their spatial reorganisation during viral maturation. The Ripley function, K(*r*), could match the expected dispersed or random distribution. However, in mature HIV-1, clustering emerges, leading to an increase in K(*r*). This correspondence can be observed through single-molecule localisation microscopy (SMLM), such as PALM or dSTORM, which provides precise nanometre-scale spatial co-ordinates of Env molecules. By applying Ripley’s K function to these datasets, it has been shown that Env proteins in immature virions do not significantly deviate from a Poisson distribution, whereas in mature virions, they form clusters that increase the local density of Env molecules. This approach is generalisable to other enveloped viruses, such as influenza and Nipah virus, which also exhibit Env glycoprotein reorganisation on their membranes. Instead of focusing on the absolute number of glycoproteins, this analysis defines clustering based on deviations from randomness, making it applicable across different viral systems regardless how many envelope glyocoproteins or spikes they package. For example, even viruses with many envelope proteins may exhibit clustered distributions, while viruses with fewer glycoproteins may display both random and strong clustering as shown in (**A**). Therefore, Ripley’s K function, when combined with SMLM data, provides a robust, quantitative framework for assessing glycoprotein clustering during viral maturation, offering insights into membrane organisation principles that extend beyond HIV-1 to a broader range of enveloped viruses.

Here, we discuss the importance of the regulation of intermolecular dynamics and concerted action of viral fusion machines to work together and generate clusters in different enveloped viruses. The human immunodeficiency virus 1 (Figure 1) (HIV-1) incorporates little amounts of envelope glycoproteins, around 10 per virus, [13,14], when compared with other enveloped viruses [13,15]. In this case, the formation of clusters might be crucial to reach the energetic requirements of fusion, as more than one Env molecule would be needed for successful completion of the fusion reaction [16]. It would also increase the probability of the HIV-1 envelope glycoprotein, Env, to engage with its main receptor, CD4, and coreceptors, CCR5 and CXCR4 [17]. The advent of single-molecule localisation microscopy (SMLM) has been instrumental in observing these processes with an unprecedented level of detail. By leveraging the latest advances in the SMLM field to their fullest potential, new insights into the mechanisms underlying viral fusion will be revealed.

### SMLM uncovers receptor clustering and their function in cells

Receptor clusters, defined as groups of receptors that aggregate in the cell membrane and act as signalling hubs, play a crucial role in enhancing and regulating cellular responses to external signals. The formation and function of these clusters are influenced by lipid order, lipid composition and actin dynamics. The concept of lipid microdomains—regions rich in cholesterol and sphingolipids—plays a crucial role in this process [18]. For instance, T-cell receptors (TCRs) cluster at the immunological synapse of T cells to recognise antigens, facilitated by the actin cytoskeleton and specific lipid compositions [8,19-21]. Epidermal growth factor receptors (EGFRs) cluster upon ligand binding, with membrane lipid order and tension playing key roles in localising these receptors for effective signalling [22]. Another example, are integrin clusters [23], crucial for cell adhesion and migration, form focal adhesions influenced by membrane fluidity in epithelial cells [24]. B-cell receptors cluster upon antigen binding, aided by actin for receptor mobility and lipid environments for signalling [25]. G protein-coupled receptor clusters, essential for processes like sensory perception, rely on actin filaments and specific lipid compositions to modulate receptor sensitivity and specificity [26,27]. As mentioned above, the regulation of these clusters is influenced by membrane fluidity, lipid order, tension, actin remodelling, providing insights into cellular signalling and potential therapeutic interventions for diseases linked to receptor clustering dysfunction.

**Figure 2:**
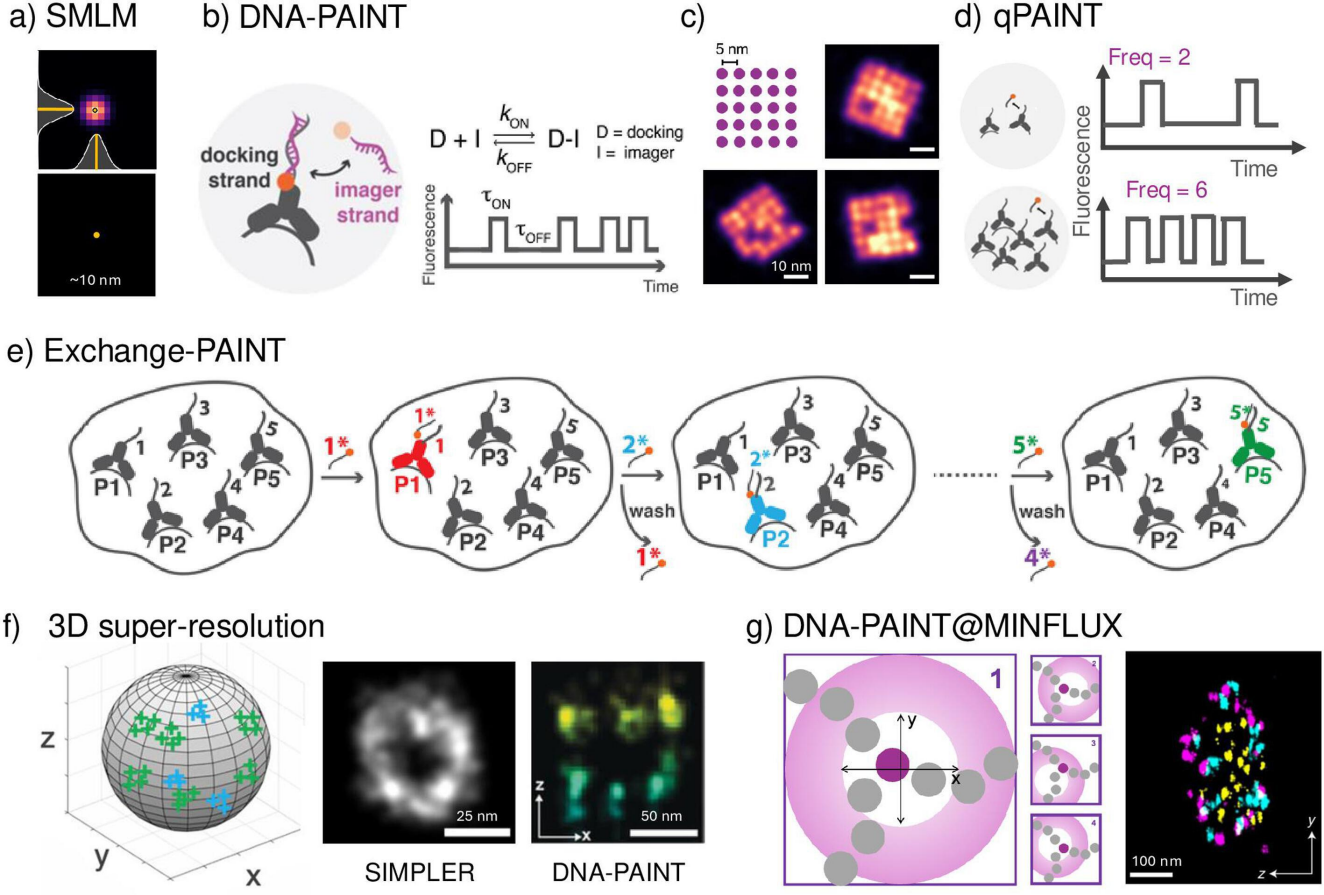
Principle of DNA-PAINT. (**A**) Basic idea of single-molecule localisation microscopy (SMLM). *Top*: Image of a single molecule that is fitted using a Gaussian curve to determine its position (*bottom* panel) with a precision ~10 nm for DNA-PAINT experiments. (**B**) DNA-PAINT involves the temporary binding of dye-labelled DNA strands (imagers) to their complementary target sequence (docking strand) attached to a protein of interest or antibodies against such protein. The transient attachment of imager strands is detected as 'blinking', depicted by the intensity vs. time trace. DNA-PAINT decouples blinking from dye photophysics while simultaneously improving the programmability and specificity of DNA molecules as imaging and labelling probes. The binding duration is exclusively determined by the stability of the generated DNA duplex and may thus be set at desire (e.g. by changing the strand length, GC content, temperature, or salinity of the imaging buffer). (**C**) Diagram of DNA origami structures featuring 25 target sites spaced 5 nm apart, representing dense biomolecular clusters. Simulated examples of DNA-PAINT high-resolution images of these structures. (**D**) Quantitative-PAINT (qPAINT) utilises the known binding dynamics between imager and docking DNA strands to determine absolute target numbers in areas where molecular resolution is not achievable. This is done by analysing the temporal changes in the fluorescence signal. If two proteins labelled with a single docking strand 'blinks' with a frequency of 2 in a certain time interval, then six proteins will blink with three times the frequency (i.e. frequency of 6). (**E**) Exchange-PAINT relies on sequential imaging of multiple targets using distinct sequences with the same fluorophore. Initially, 1* imager strands in solution bind to their complementary target sequence, P1. After the first imaging round, 1* imagers are washed out and replaced by 2* imagers to target P2. This process is repeated for the remaining target cycles, assigning pseudocolours to each imaging round. (**F**) *Left*: Schematic illustrating 3D single-molecule localisations on a virus. *Centre*: An average (*n* = 8) microtubule profile using microscopy photometric z-localisation with enhanced resolution (SIMPLER). This figure has been adapted from [30] with permission from *Nature Communications*, Springer Nature, copyright 2021. *Right*: Overview image of NUP96-Halo imaged using 3D DNA-PAINT showing the organisation in nuclear and cytoplasmic rings. Scale bar: 50 nm. This figure has been adapted from [31] with permission from Angewandte Chemie, copyright 2019. (**G**) *Left*: An excitation laser beam (pink) is shaped by a vortex-phase mask forming a doughnut intensity spot in the focal plane of the objective lens. The intensity of the beam is modulated and deflected such that its central zero is sequentially placed at the four focal plane positions. The fluorescence photons detected by pinhole detector for each donut position are used to extract the position of the molecule of interest. *Right*: Cross-section of a mitochondrial tubule shown in of fixed U2OS TOM70-Dreiklang cells imaged with DNA-PAINT-MINFLUX. This figure has been adapted from [32] with permission from *Nature Methods*, Springer Nature, copyright 2022. PAINT, point accumulation for imaging in nanoscale topography.

Super-resolution fluorescence microscopy techniques [28] have been instrumental in defining and understanding receptor clusters. According to Abbe’s criteria established in 1873, spatial resolution is limited by the diffraction of light to a few hundred nanometres. As a result, traditional microscopy techniques could not achieve the resolution needed to observe the fine details of receptor organisation at the nanoscale. However, SMLM, such as direct stochastic optical reconstruction microscopy [29] (dSTORM) and photoactivated localisation microscopy [28] (PALM), has overcome this limitation by exploiting the fact that the centre position of a single molecule’s image can be determined with much higher accuracy than the size of the image itself (Figure 2A).

In practice, these techniques rely on the temporal isolation of individual fluorescent molecules by switching between a dark and a bright state, allowing subsequent spatial localisation where the impact of the Abbe criteria is reduced, allowing the reconstruction of super-resolution images. The sparse set of point-spread functions is observed on a camera whose centres can be determined with nanometric precision. These coordinates are saved, the fluorophores or fluorescent proteins are photobleached or switched back to the dark state, and a new sub-set of fluorescent molecules is recorded [33]. Thanks to the visualisation of individual molecules with nanometre precision, the observation of formation, dynamics and dissociation of clusters is now possible. For instance, super-resolution microscopy has been used to study TCRs at the immunological synapse [34], revealing how they cluster and interact with the actin cytoskeleton during antigen recognition. Similarly, it has been crucial in characterising the clustering of EGFRs, showing how membrane lipid order and tension influence their spatial distribution upon ligand binding [35]. Neurotransmitter receptors like N-methyl-D-aspartate (NMDA) receptors have also been studied using these techniques, providing detailed insights into their organisation at synaptic sites and their interactions with scaffolding proteins and lipid environments [36]. In summary, super-resolution microscopy has revealed the intricate details of receptor interactions with the actin cytoskeleton and lipid microdomains in many biological examples, enhancing our understanding of how biophysical parameters affect receptor clustering. These insights have been paramount for elucidating the mechanisms underlying cellular signalling and the regulation of receptor functions, paving the way for potential and future targeted therapeutic strategies in diseases associated with receptor clustering dysfunction.

### SMLM uncovers fusogen clustering and their function in cells

In the context of single enveloped viruses, Yuan and colleagues [37] highlighted the critical role of CD4 clustering in facilitating HIV-1 engagement, showing that pre-clustered CD4 molecules enhance the efficiency of Env binding and subsequent viral entry. CD4 clustering is thought to increase the local concentration of receptors, thereby improving the probability of Env-receptor interactions and promoting conformational changes necessary for fusion. Beyond CD4, other studies have demonstrated that upon HIV-1 Env binding, CD4 and coreceptors (CXCR4 or CCR5) undergo significant reorganisation within the plasma membrane [9]. For instance, single-molecule and super-resolution imaging studies have shown that Env engagement induces dynamic coalescence of CD4 and coreceptors into signalling platforms, which might enhance viral fusion efficiency [17]. This clustering is also associated with actin remodelling and lipid raft reorganisation, processes that are linked to viral entry and infection. Disrupting these clusters, for example, through pharmacological inhibitors or membrane-modifying agents such as SERINCS [38,39], has been shown to impair HIV-1 fusion, highlighting the functional relevance of receptor organisation during viral entry.

Similar to HIV-1, receptor clustering plays a crucial role in the entry of other enveloped viruses, such as Influenza A virus (IAV) and the paramyxovirus Nipah virus (NiV). For IAV, sialylated glycan receptors are not randomly distributed on the plasma membrane but rather form nanoclusters that can enhance viral binding and entry [40]. Studies using super-resolution microscopy have demonstrated that IAV haemagglutinin (HA) preferentially interacts with specific glycan-enriched microdomains, which facilitate receptor engagement and endocytosis [41]. The spatial organisation of these receptors affects viral tropism and susceptibility to infection. Similarly, for NiV, ephrin-B2 and ephrin-B3 receptors, which are essential for viral entry, have been shown to localise within specific membrane domains [42,43]. Their clustering enhances fusion glycoprotein (GP) interactions and might facilitate viral-host membrane fusion [44]. Disrupting these receptor clusters, whether through pharmacological means or genetic modifications, has been found to reduce viral entry efficiency [45], highlighting the importance of receptor organisation in infection dynamics across multiple viral families.

### The particular case of HIV-1 maturation and its dependence on Env clustering for viral entry

HIV-1 maturation is a crucial step in the viral life cycle that transforms the virus from an immature, non-infectious particle into a mature, infectious one [46]. This process is driven by the viral protease, which cleaves the Gag and Gag-Pol polyproteins into their functional components, resulting in structural reorganisation such as core condensation. Env glycoproteins, existing as trimeric spikes on the viral envelope, play a pivotal role in the entry process by facilitating CD4 receptor and coreceptor (CCR5/CXCR4) binding and starting a number of Env conformational changes that will culminate in membrane fusion [47]. The clustering of Env spikes might enhance the avidity of binding to host cell receptors [11], thereby improving the virus’s infectivity, and also contribute to immune evasion by shielding critical epitopes from neutralising antibodies as discussed in [48]. Env clustering involves cooperation among multiple Env trimers to achieve the necessary energy for successful viral and host membrane fusion. During this fusion process, Env undergoes significant conformational changes, particularly in the gp41 subunit, which forms a helical stalk that drives both the viral and host membranes together. The fusion reaction occurs through a hemifusion intermediate [49], where the outer leaflets of the viral and cellular membranes merge before the formation of a complete fusion pore. Biophysically, this involves overcoming membrane tension and accounting for the composition of both viral and host cell membranes, which influences the efficiency of membrane fusion itself [50].

Despite the importance of Env in the fusion process, HIV-1 incorporates relatively few Env molecules into its membrane compared with other viruses with similar fusogens, necessitating the cooperative action of several Env trimers to meet the energy requirements for fusion as referred above. This understanding is critical for HIV vaccine design, as vaccines need to elicit broadly neutralising antibodies targeting conserved and accessible Env epitopes that physically impede Env to proceed across the fusion reaction. For instance, immunogens that mimic the native trimeric structure of Env are being explored to induce effective immune responses. Thus, the relationship between HIV-1 maturation, Env clustering and membrane fusion mechanics is integral to the virus’s infectivity and immune evasion strategies [51]. In this context, dSTORM has recently revealed that Serine incorporator protein 5 inhibits HIV-1 infectivity by disrupting the clustering of Env on virions, which sensitises the virus to neutralising antibodies and accelerates Env function loss [52]. Interestingly, dSTORM has also been instrumental in showing that human interferon-induced transmembrane (IFITM) proteins reduce HIV-1 infectivity not by affecting Env clustering, but by decreasing Env processing and incorporation into virions, indicating that IFITM proteins restrict HIV-1 through mechanisms other than modifying Env clustering [53]. Understanding these distinct mechanisms of viral restriction can provide valuable insights for developing more effective therapeutic and preventive strategies against HIV-1.

Modulating molecular clustering in the plasma membrane is challenging due to the limitations of conventional pharmacological agents. Many commonly used drugs, such as methyl-β-cyclodextrin and cytochalasin B, can disrupt membrane organisation by extracting cholesterol or interfering with the actin cytoskeleton, respectively [54]. However, these compounds often have cytotoxic effects, induce off-target changes, or lack specificity, making them unsuitable for precise control over clustering [55]. Recent advancements in nanotechnology have introduced DNA origami as a potential alternative for modulating clustering externally. DNA origami structures can be designed to interact with membrane components in a highly programmable manner, allowing for spatially controlled clustering without the toxic side effects associated with chemical treatments [56]. Some studies have demonstrated the use of DNA origami scaffolds to organise membrane proteins or lipid domains [57], offering a promising tool for studying membrane dynamics with improved specificity and minimal cellular perturbation.

### Examples of enveloped viruses where fusogens cluster to achieve efficient membrane fusion

Several enveloped viruses rely on the clustering of fusogens to achieve efficient membrane fusion, with various experimental studies substantiating this phenomenon. For example, the influenza virus utilises HA proteins that cluster on both the cellular membrane [58] and the viral envelope to facilitate binding to sialic acid receptors on host cells [59], followed by membrane fusion. Similarly, herpes simplex virus (HSV) employs glycoproteins gB, gD, gH, and gL, which cluster and co-operatively mediate membrane fusion [60]. Experiments using viral mutants deficient in these glycoproteins demonstrated that disrupting this cluster formation significantly impairs viral entry into host cells.

The respiratory syncytial virus relies on its F (fusion) protein, which also clusters on the viral surface. Structural and biophysical analyses, including cryo-electron microscopy, have revealed that these clusters undergo significant conformational changes, driving membrane fusion and infection [61]. In the case of the Ebola virus, the glycoprotein (GP) clusters and facilitates viral entry through interactions with host cell receptors [62]. Experimental studies using fluorescently labelled GP have shown that clustering enhances the virus’s ability to fuse with and enter host cells [63].

Additionally, in NiV, the G (attachment) and F (fusion) proteins, which also cluster to mediate viral entry [64]. The G protein binds to the ephrin-B2 or ephrin-B3 receptors of the host cells, leading to conformational changes in the F protein, promoting membrane fusion. Research has demonstrated that clustering of the G and F proteins is crucial for efficient fusion [42]. Indeed, mutational analyses disrupting the interaction and clustering of these proteins have shown a marked decrease in fusion activity, underscoring their co-operative function. Additionally, co-immunoprecipitation assays and live-cell imaging studies have provided evidence that both G and F proteins must be present in specific stoichiometries and proximities to facilitate efficient membrane fusion [43]. These examples collectively underscore the critical role of fusogen clustering in the membrane fusion process of enveloped viruses, demonstrating that this structural organisation is essential for viral infectivity.

### Overview and future directions

Receptor clusters in cells and the grouping of viral fusogens exhibit similar traits and evolutionary parallels, driven by shared principles of enhancing functional efficiency and specificity. Both mechanisms rely on spatial organisation to facilitate effective interactions: receptor clusters enhance signal transduction by bringing together key molecules, while viral fusogens optimise membrane fusion by increasing efficiency in the fusion reaction. This dynamic spatial organisation probably offers selective advantages. We speculate that these similarities could lead to evolutionary convergence. Equally important is the regulation of biophysical aspects of membrane composition, including lipid order and tension, which can significantly influence the grouping of receptors and fusogens. Membrane lipid order and tension can modulate the fluidity and curvature of the membrane, affecting how these molecules cluster and function. For instance, tightly packed lipids may promote the stable clustering of receptors, while specific lipid compositions in viral envelopes can enhance fusogen activity. The possibility to experimentally observe viral protein cluster formation using single-molecule-based super-resolution fluorescence microscopy in isolated viruses or infected cells would open new insights into the complex regulation of viral fusion.

In the context of protein clustering of receptors at the surface of cells, researchers have swiftly embraced cutting-edge ultra-resolved, quantitative, and highly multiplexed 2D and 3D super-resolution imaging techniques. These advancements have enabled the observation of receptor clusters at the nanoscale with unprecedented clarity, unveiling crucial details that underpin their functional roles [65,66]. Such insights are pivotal for the development of targeted therapies and for comprehending fundamental cellular processes, highlighting the intricate relationship between molecular organisation and membrane biophysics. To deepen our understanding of how fusogen clustering influences virus entry, the virology community must stay abreast of the latest advancements in SMLM. Notably, DNA-PAINT (point accumulation for imaging in nanoscale topography) [67] relies on the transient binding interaction between two short single-stranded DNAs: one fluorescently labelled and freely diffusing in solution (imager strand), and the other chemically coupled to an antibody (docking strand) [68]. As for PALM and dSTORM, a fluorescent signal appears as a diffraction-limited spot, but this time when the imager binds to the docking strand and is immobilised, the signal disappears when the imager dissociates from the docking (Figure 2B). The advantage of DNA-PAINT compared with other SMLM approaches is that the imager strands are continuously replenished, making DNA-PAINT immune to photobleaching and allowing the same target protein to be detected multiple times. Moreover, because the fluorophore does not need to be inherently switchable or activatable, it allows the use of bright and stable fluorophores. Recent progress in DNA-PAINT imaging has even led to the development of dye-quencher or dye-dye self-quenching imager probes, which greatly minimise background fluorescence, enhance brightness, and boost spatial resolution while enabling faster imaging speeds [69]. These innovations further enhance DNA-PAINT’s localisation precision, making it superior to other SMLM methods.

DNA-PAINT can routinely achieve sub-5 nm localisation precision, with effective resolutions in the range of 10 nm. Recent advancements in sequential imaging using this technique have demonstrated Ångström-level resolution [70]. The high level of nanometre accuracy attained by DNA-PAINT has proven capable of imaging individual molecular targets on 2D synthetic samples that simulate dense biomolecular nanoclusters [71] (Figure 2C). Moreover, due to the predictable binding kinetics between imager and docking strands, DNA-PAINT can provide accurate quantitative information by correlating the frequency of single-molecule events with the underlying number of labelled molecular targets, a technique known as qPAINT [72] (Figure 2D). Another competitive advantage of DNA-PAINT over other SMLM techniques is its capability for highly multiplexed super-resolution imaging (Figure 2E). For instance, it has recently demonstrated 30 colour imaging [73] in DNA-origami and fixed neuron samples with 3D super resolution [74]. This contrasts sharply with the challenges faced by other methods, where the use of limited probes with overlapping spectra, potential colour crosstalk, and different mounting media often restricts multicolour super-resolution imaging to a maximum of three colours. This is possible by sequential washout and exchange of imager strands (i.e. Exchange-PAINT, Figure 2E); key to understanding protein interactions in the context of virology and immunology.

In combination with advances in 3D SMLM imaging, including supercritical angle fluorescence [75], photometric analysis of defocused images [76], and supercritical illumination microscopy photometric z-localisation with enhanced resolution [30] (SIMPLER), DNA-PAINT is capable of achieving 10 nm isotropic resolution in 3D (Figure 2F). Notably, the combination of DNA-PAINT with minimal fluorescence photon fluxes (MINFLUX) nanoscopy has proven capable of even greater enhancement in 3D resolution, with isotropic localisation precision below 2 nm [32] (Figure 2G). Imaging with this level of detail is a significant advancement in the field, and we expect that in the future, these capabilities can allow us to truly observe the molecular distribution of the fusogens on the surface of viruses, enabling researchers to uncover intricate details of their spatial organisation that drive their function. The drawbacks for this approach are that often imaging is limited to fixed samples and slow imaging speeds (to accumulate enough localisations per molecule, thousands of frames are needed, which in practice means acquisitions of hours). Also, this approach is combined with total internal reflection microscopy, which implies illumination profiles restricted to 100–200 nm above the coverslip (for viruses and membranes that is ideal); but other biological processes beyond the membrane might be difficult to image.

In summary, the convergence observed between receptor clusters in cells and viral fusogen groupings underscores their shared evolutionary principles in enhancing functional efficiency through spatial organisation. Advances in single-molecule-based super-resolution imaging, particularly through techniques like DNA-PAINT, offer unprecedented clarity and multiplexing capabilities crucial for studying these phenomena. These advancements are poised to revolutionise our understanding of viral entry mechanisms and cellular processes, promising deeper insights into the molecular distribution and functional dynamics of fusogens on virus surfaces.

**Perspectives**

- We stress the importance of virus–cell fusion and how it is executed by groups or clusters of viral surface glycoproteins. Single-molecule localisation microscopy (SMLM) allowed us to visualise intermolecular dynamics on the viral surface. Many envelope viruses contain envelope glycoprotein clusters, and this suggests their importance in entry.
- These dynamics are important in many scenarios because surface glycoproteins need to work in concert to attain the high energetic requirements to culminate the merging of both the host and the viral membrane. We hypothesise that these envelope glycoprotein clusters might be crucial for entry and fusion and also to avoid immunisation by broadly neutralising antibodies.
- We stress its importance as a common strategy during the viral fusion reaction for different viruses, including influenza, Nipah or human immunodeficiency virus (HIV). Future developments in advanced microscopy will help delineate strategies to fully understand surface glycoprotein co-operation during fusion.

## Abbreviations

DNA-PAINT: DNA point accumulation for imaging in nanoscale topography
EGFRs: Epidermal growth factor receptors
GP: glycoprotein
HA: IAV haemagglutinin
HIV-1: human immunodeficiency virus 1
HSV: herpes simplex virus
IAV: Influenza A virus
IFITM: human interferon-induced transmembrane
MINFLUX: minimal fluorescence photon fluxes
NMDA: Neurotransmitter receptors like N-methyl-D-aspartate
NiV: Nipah virus
PALM: photoactivated localisation microscopy
SIMPLER: supercritical illumination microscopy photometricz-localisation with enhanced resolution
SMLM: single-molecule localisation microscopy
TCRs: T-cell receptors
dSTORM: direct stochastic optical reconstruction microscopy
